# The “Worktivity” mHealth intervention to reduce sedentary behaviour in the workplace: a feasibility cluster randomised controlled pilot study

**DOI:** 10.1186/s12889-021-11473-6

**Published:** 2021-07-18

**Authors:** Aoife Stephenson, Matias Garcia-Constantino, Marie H. Murphy, Suzanne M. McDonough, Chris D. Nugent, Jacqueline L. Mair

**Affiliations:** 1 Centre for Exercise Medicine, Physical Activity and Health, Ulster University, Shore Road, Co. Antrim BT37 0QB Newtownabbey, UK; 2 School of Physiotherapy, Royal College of Surgeons in Ireland (RCSI) University of Medicine and Health Sciences, 123 St Stephens Green, Dublin, Ireland; 3 School of Computing, Ulster University, Shore Road, Newtownabbey, Co. Antrim BT37 0QB UK; 4 School of Health Sciences, Ulster University, Shore Road, Newtownabbey, Co. Antrim BT37 0QB UK; 5 Future Health Technologies, Singapore-ETH Centre, Campus for Research Excellence And Technological Enterprise (CREATE), Singapore, Singapore

**Keywords:** Sedentary lifestyle, Occupational health, Office work, Digital health, Health behaviour, Mobile apps, Sit-stand work desk

## Abstract

**Background:**

Office work generally consists of high amounts of sedentary behaviour (SB) which has been associated with negative health consequences. We developed the “WorktivIty” mobile app to help office workers reduce their SB through self-monitoring and feedback on sedentary time, prompts to break sedentary time, and educational facts. The aim of this paper is to report the feasibility of delivering the Worktivity intervention to desk-based office workers in the workplace setting and describe methodological considerations for a future trial.

**Methods:**

We conducted a three-arm feasibility cluster randomised controlled pilot study over an 8-week period with full time-desk based employees. Clustered randomisation was to one of three groups: Worktivity mobile app (MA; *n* = 20), Worktivity mobile app plus SSWD (MA+SSWD; *n* = 20), or Control (C; *n* = 16). Feasibility was assessed using measures of recruitment and retention, intervention engagement, intervention delivery, completion rates and usable data, adverse events, and acceptability.

**Results:**

Recruitment of companies to participate in this study was challenging (8% of those contacted), but retention of individual participants within the recruited groups was high (81% C, 90% MA + SSWD, 95% MA). Office workers’ engagement with the app was moderate (on average 59%). Intervention delivery was partially compromised due to diminishing user engagement and technical issues related to educational fact delivery. Sufficient amounts of useable data were collected, however either missing or unusable data were observed with activPAL™, with data loss increasing at each follow up time point. No serious adverse events were identified during the study. The majority of participants agreed that the intervention could be implemented within the workplace setting (65% MA; 72% MA + SSWD) but overall satisfaction with the intervention was modest (58% MA; 39% MA + SSWD).

**Conclusions:**

The findings suggest that, in principle, it is feasible to implement a mobile app-based intervention in the workplace setting however the Worktivity intervention requires further technical refinements before moving to effectiveness trials. Challenges relating to the initial recruitment of workplaces and maintaining user engagement with the mHealth intervention over time need to be addressed prior to future large-scale implementation. Further research is needed to identify how best to overcome these challenges.

**Supplementary Information:**

The online version contains supplementary material available at 10.1186/s12889-021-11473-6.

## Introduction

It is well established that high levels of sedentary behaviour (SB) are associated with a range of health concerns. A recently updated systematic review shows that high amounts of SB increase the risk for all-cause, cardiovascular disease (CVD) and cancer mortality, as well as incidence of CVD, cancer, and type 2 diabetes [1]. Another overview of 18 systematic reviews suggests that high levels of SB are unfavourably associated with cognitive function, depression, function and disability, physical activity levels, and physical health-related quality of life [2]. These findings have important public health implications and suggest that individuals should avoid high levels and prolonged bouts of SB [2].

Office work is generally characterised by prolonged periods of SB and contributes significantly to the overall sedentary time of workers [3]. Sedentary activities have been shown to comprise 65–82% of time at work in industrialised countries [3–5] with a large proportion (54–77%) of office workers’ total daily sitting time occurring during their working day [5–7]. Due to this high prevalence, occupational sitting has become an emergent workplace health issue [8].

Regularly interrupting SB with light-intensity physical activity can improve several cardiometabolic, musculoskeletal and psychological health outcomes [9–12]. However, implementing interventions that involve frequent physical activity breaks in the workplace setting remains a challenge. Common barriers to reducing SB at work include the prioritisation of work tasks, “sitting-centred” office design, reliance on desk-based information technology, fear of being judged as “avoiding work” when not sitting, and a lack of knowledge on how and why to reduce SB [13]. Threats to productivity posed by workplace SB interventions are also of particular concern to both employees and employers [13] but little is known about the consequences of SB on office workers’ well-being and work performance [14]. Therefore, although SB reduction can improve health outcomes, behaviour change is unlikely to occur in the workplace setting unless we provide education on the negative health consequences of SB, create environments that facilitate standing and moving at work, encourage cultures that are supportive of alternative ways of working, and deliver evidence that will convince employers to invest in SB reduction strategies.

It has been suggested that interventions including environmental restructuring may be promising in reducing occupational SB [15]. Specifically, there is evidence to support the use of sit-stand work desks (SSWD) in conjunction with other behavioural intervention approaches such as management support, health coaching, goal setting, self-monitoring, use of prompts and problem solving, workplace policy changes, and informational components [16–18]. Implementing SSWDs can, however, be costly and in most countries there is currently no requirement to offer these to staff from an occupational health perspective. Therefore, while interventions involving SSWDs appear effective, it is important to establish alternative strategies to reduce SB that are relevant to a wide range of occupations and working environments.

Given the prevalence of digital devices in office settings, technology-based strategies have the potential to play an important role here. Previous randomised controlled trials [19, 20] suggest that multicomponent interventions comprising of digital tools (computer prompts, reminder emails) in conjunction with a SSWD may help to reduce occupational sitting time. However, beyond computer -based prompts, little is known about how digital interventions can be used to reduce SB in office workers and how the behaviour change potential of technology can be supplemented by environmental restructuring approaches such as SSWDs. Mobile health (mHealth) approaches can broaden the reach and scale of behaviour change interventions at a low cost, be highly personalised, and deliver information in a flexible way that is engaging and rewarding [21, 22]. Desk-based office workers potentially have much to gain from mHealth interventions for SB reduction, yet little is known about the impact of this technology in a “real-world” workplace context.

We attempted to address this challenge by developing a novel, theory based, and user informed mobile app intervention - “Worktivity” - designed to reduce occupational SB. In line with guidelines on the development and evaluation of complex interventions [23], the Worktivity intervention was developed using a four-phase iterative approach based on formative research findings [24]. We then progressed to feasibility and pilot testing of the intervention to better understand “how” and “why” Worktivity might work in practice and to inform a future definitive trial on its effectiveness. The specific aim of this paper is to report the feasibility of delivering the Worktivity intervention to desk-based office workers in the workplace setting and describe methodological considerations for a future trial. Specifically, we describe the recruitment process and retention of participants, intervention delivery within the workplace setting, engagement with the intervention, participation completion rates and the amount of usable data collected, any adverse events that occured, and participant satisfaction.

## Methods

### Study design

**Fig. 1:**
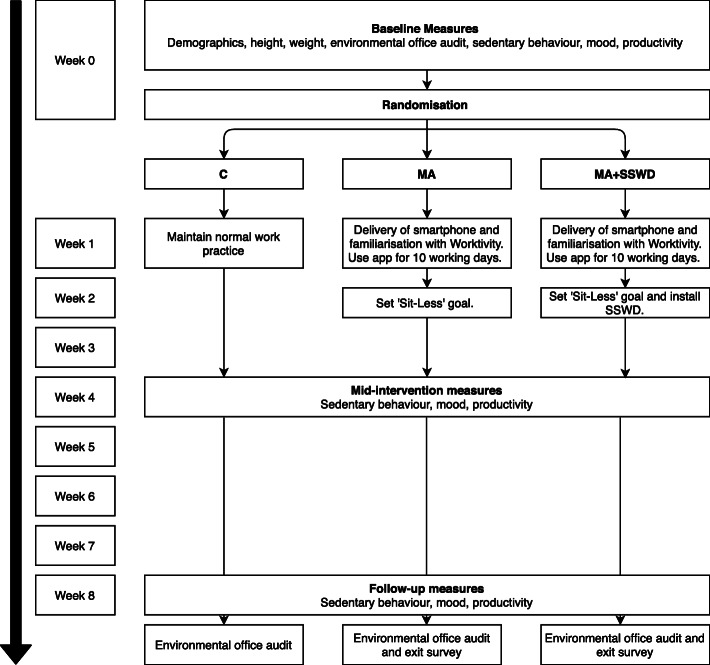
Study flow

The study design was a three-arm feasibility cluster randomised controlled pilot study. The study flow is outlined in Fig. 1.

### Sample size

As this was a feasibility study, a sample size calculation was not necessary [25]. Instead, we aimed to recruit 20 participants across three worksites (total *n* = 60) based on previous feasibility studies with similar aims [26, 27], resource considerations and the recommendation for feasibility studies to have at least 50 participants [28].

### Participants

E-mail invitations were sent to managers in companies with desk-based office workers across Northern Ireland (*N* = 39). Three companies were recruited to the study and managers within each of the interested companies passed on study information to staff. Approximately one week later, researchers visited the worksites to screen for individual eligibility and obtain informed consent.

#### Inclusion criteria

- Company office based in Northern Ireland with at least 20 full-time office workers
- Predominantly desk-based office workers (self-reported > 50% seated working hours)
- Working full time hours (> 30 h per week)
- Aged 18–65 years

#### Exclusion criteria

- Pregnancy (as their activity/SB may be pregnancy related)
- Planned absence of > five consecutive working days over the course of the intervention
- Unable to read and understand English (since the intervention was only provided in English)
- Non-ambulant or severely incapacitated with existing conditions restricting ability to stand/move
- Currently participating in a study to reduce SB/increase physical activity

### Randomisation

Cluster randomisation was used to assign worksites to one of three arms:

1. Mobile phone app (MA)
2. Mobile phone app plus height adjustable work desk (MA + SSWD)
3. Control (C)

The three worksites and the three allocations were concealed in sealed opaque envelopes. An independent researcher, with no links to this project, selected one envelope which contained the worksite name and another envelope which contained the assigned group. The worksite was matched with the corresponding group allocation. This was conducted twice more until randomisation was complete.

### Blinding

Blinding of participants and study personnel to group allocation was not possible, due to the nature of the study. Participants in the control group were advised to maintain normal work practices for the study duration.

### Intervention

Those in the MA and MA + SSWD groups were given a mobile phone and charger (Motorola XT1068 Moto G (Second Generation)) with the Worktivity app installed. Participants were provided with information on how to use the app and asked to use it for study purposes only.

**Fig. 2:**
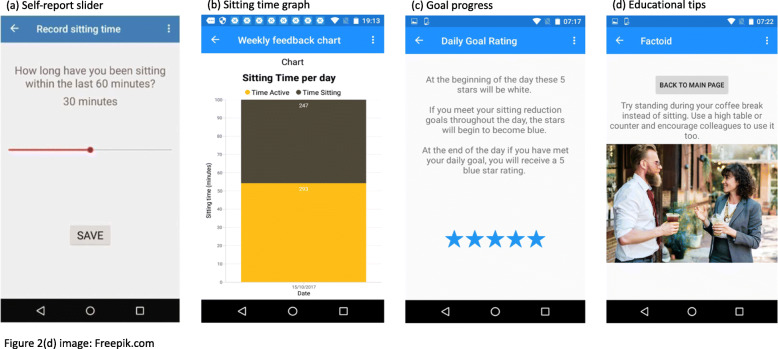
Screenshots of the Worktivity app features

The Worktivity app is described in detail elsewhere [24]. Briefly, the app targets SB change by allowing users to self-monitor their SB, view feedback on their SB in real-time, set “sit-less goals”, receive prompts/reminders to meet their goals, visualize goal progression, and receive educational tips on how to reduce SB. The self-monitoring feature of the app uses an Ecological Momentary Assessment (EMA) approach to prompt users to log their SB at work at hourly intervals throughout the work day using a quick and simple self-report slider (Fig. 2a). The feedback feature presents users with a personalised sitting time graph summarising the data by day and by week, to be viewed at any time (Fig. 2b). The goal setting feature allows users to specify by how much time they want to reduce their sitting at work. The app then calculates a reminder to meet their goal based on their self-monitoring data. Progress towards goals are visualised through a points-earning system (Fig. 2c). Educational tips are delivered at the end of each workday and provide information on the health consequences of sitting and potential strategies that could be used to reduce occupational SB (Fig. 2d).

Both intervention arms used the app to self-monitor SB over a two-week baseline period (10 work days). Participants were then guided by the researchers on how to set a personalized “sit-less” goal within the app. Goals were based upon the participants’ baseline self-monitored sitting time (available through the app feedback function), and recommendations on workplace SB (2 h/day of standing and light PA during working hours, progressing to 4 h/day) [29]*.* Based on the “sit-less” goal and the continued real time self-monitoring, a prompt with advice to break sitting was delivered if sitting time was too high. Once the goal setting feature was enabled, the original self-monitoring and feedback aspects continued, but with the addition of a goal visualisation feature and educational tips and facts section. Once participants logged their eighth and final hourly sitting time each day they received an educational fact/tip to assist with reducing SB.

Following goal setting, the MA + SSWD intervention arm were provided with a SSWD (Workfit-T, Ergotron, MN, USA) to place on their existing work desk. Researchers installed the desks and instructed participants on correct usage. The SSWD allowed the user to alternate between sitting and standing postures at their desk, giving further opportunity to reduce occupational SB. Participants used the app for a further six weeks (30 working days) (Fig. 1).

### Outcome measures

Socio-demographic characteristics, including educational level, occupation, and income bracket, were obtained via questionnaire at baseline in order to describe the sample recruited. Height was measured in meters using a stadiometer (Seca stadiometer 220/222, Hamburg, Germany) and weight was measured in kilograms using digital scales (Seca scales 899, Hamburg, Germany) in a private room within each worksite at baseline. Body mass index (BMI) was then calculated using the formula: weight (kg)/height (m)^2^. Sedentary behaviour, productivity and mood were measured during each measurement period (basline, mid-intervention, and follow-up) as detailed in Fig. 1.

Sedentary behaviour was assessed using a thigh-worn accelerometer (activPAL™, PAL Technologies, Scotland). Participants were given an activPAL™ device and instructions on how to use it. The activPAL™ was secured to the skin at the anterior mid-line of the thigh using hypoallergenic adhesive patches and was to be removed during bathing and swimming. For ethical reasons, participants were also informed of the risk of skin irritation with adhesive patches and that they should remove the patch and attach the activPAL™ to the other thigh or stop wearing the activPAL™ if skin irritation occurred. Participants were asked to keep a diary to log activPAL™ device removal and reasons why, to record the times they got up, went to bed, started and finished work, and note any other comments related to the study.

Productivity was measured using EMA. This approach was chosen over a questionnaire based measure to minimise recall bias and maximise ecological validity [30]. On each of the five work days during each measurement period, participants were sent the following message, via text or email (based on their preferred delivery method), towards the end of their work day (approximately between 15.30–16.30 h): “On a scale from 0 to 10, where 0 is the lowest and 10 is the highest, how would you rate your work productivity today [insert date]? (Please reply with the number that best corresponds to your productivity today [insert date])”. If the participant did not respond on that day, a follow up message was sent the morning of the next working day. A similar procedure has been used to assess productivity in a workplace setting [31].

Mood was assessed using the Brunel Mood Scale (BRUMS) [32, 33] which has been validated for use in healthy adult populations [34]. BRUMS is a 24-item questionnaire of simple mood descriptors divided across six subscales: anger, confusion, depression, fatigue, tension, and vigour. At each measurement time point participants were asked to indicate the extent to which they had experienced the feelings described by the 24 mood descriptors over the last week. Responses were recorded using a five-point Likert scale, where ‘0’ = ‘Not at all’, ‘1’ = ‘A little’, ‘2’ = ‘Moderately’, 3 = ‘Quite a bit’, and ‘4’ = ‘Extremely’. The participants were asked to complete the form when they removed the activPAL™ after each seven-day measurement period.

Additionally, an environmental audit was conducted at baseline and follow-up to assess the context in which the intervention was delivered and any unintended environmental changes that may have influenced the intervention (Additional file 1).

The feasibility outcomes of interest are outlined in Table 1. Various data sources were used to gather information on feasibility throughout the study including researcher notes and mobile app analytics. In addition, an exit survey consisting of both closed and open ended questions was conducted to explore the participants’ experience and satisfaction with the intervention.

**Table 1:** Overview of intervention feasibility and preliminary response outcome measures

Outcome	Description	Evaluation method	Evaluation timepoints	Groups
**Trial feasibility**				
Recruitment and retention	How well were participants recruited to, and retained within, the study?	Recruitment and retention logs	Throughout intervention	MA MA + SSWD
Completion rates and usable data	How appropriate were data collection methods and were there instances of missing data?	Completion and usable data logs, activPAL™ diary and analysis	Baseline, mid-intervention and follow-up	MA MA + SSWD C
Adverse Events	What unexpected issues arose during the trial?	Researcher notes Exit survey	Throughout intervention	MA MA + SSWD C
Acceptability	How did satisfied were the participants to use the intervention?	Exit survey	Post follow-up	MA MA + SSWD
**Technical Feasibility**				
Engagement	Did participants engage with and respond to the app prompts to self-monitor sitting time as intended?	App analytics Exit survey	Throughout intervention and post follow-up	MA MA + SSWD
Delivery	Were intervention features and activities implemented as intended?	Researcher notes Exit survey	Throughout intervention and post follow-up	MA MA + SSWD

*C* control group, *MA* mobile app group, *MA + SSWD* mobile app plus sit-stand work desk group

### Data analysis

Analyses reported here focus on the feasibility of delivering the Worktivity intervention to desk-based office workers in the workplace setting. Intervention feasibility is summarized narratively and descriptively. Quantitative data were analysed using SPSS Version 23.0 and Microsoft Excel 2010 and reported as descriptive statistics (mean, standard deviation, percentages). If missing data occurred, the missing data were not imputed. Qualitative short answer responses from the exit survey were coded and summarised into themes.

Enagagement with the app prompts was assessed using data collected by the app. The number of prompts acknowledged, the number of missed prompts, the response rate (percentage of timely vs. delayed) and mean response time were extracted. The *acknowledgement* of a prompt meant that the participant opened the notification and reacted by self-reporting their sitting time during the previous hour. The *response rate* to the prompt meant the number of times the participants recorded their sitting time quickly (< 1 min after prompt delivery) or with a delay (> 1 min after prompt delivery) reported as a percentage of total acknowledgements.

The activPAL™ data were analysed using an algorithm [35] in STATA (STATA IC Version 15.0). To maximise the amount of data available for analysis from the working week (five weekdays) from our small sample size, two valid weekdays from each of the three measurement periods were required. Days were classed as non-wear (invalid) if they met any of these criteria: limited variation in activities (⩾95% of waking wear in any one activity); limited stepping (< 500 steps); or, limited waking wear time (< 10 h) [35]. Two valid days of data with ≥4 h wear time at work (e.g. between 09:00–17:00) were required for workday analysis. Output variables included number of valid days, average wear-time, average daily time spent sitting, standing and stepping, average number of sit-to-stand transitions per day, average number of prolonged (> 60 min) sitting bouts.

The output was visually checked against diary data for unusual episodes. If no sleep was identified in the output file, but recorded in the diary, the data for that day was removed from the analysis. If the removed day’s corresponding work hours met the specific workday wear-time criteria, the data was used in the workday analysis. In order to isolate time spent at work, the process recommended by Edwardson et al. [36] was used. Events (bouts of sitting, standing, stepping) were included if ≥50% of that bout was within the period of interest (09:00–17:00).

## Results

### Demographic analysis

Fifty-six participants (61% male) were cluster randomised to the control (*n* = 16), MA (*n* = 20) or MA + SSWD (*n* = 20) arms. Participant characteristics are presented in Table 2.

**Table 2:** Participant demographics

		C (***n*** = 16)	MA (***n*** = 20)	MA + SSWD (***n*** = 20)	Total (***n*** = 56)
Age (years)		37.9 ± 10.6	36.7 ± 10.2	33.6 ± 8.9	35.9 ± 9.9
Sex (F/M)		9 / 7	4 / 16	9 / 11	22 / 34
Height (m)^a^		1.67 ± 0.10	1.75 ± 0.08	1.74 ± 0.10	1.73 ± 0.10
Weight (kg)		84.0 ± 15.3	82.5 ± 13.1	78.6 ± 16.6	81.5 ± 15.0
BMI (kg/m^2^)^a^		30.2 ± 6.4	27.0 ± 3.7	25.8 ± 4.1	27.4 ± 4.9
Hours worked per week		38.22 ± 1.3	39.5 ± 3.5	39.9 ± 3.7	39.3 ± 3.2
Education (n)	Level 7 (e.g. Master’s Degree)	9	5	7	21
	Level 6 (e.g. Bachelor’s Degree)	6	10	11	27
	Level 5 (e.g. HND, DipHE)	0	1	0	1
	Level 4 (e.g Diploma, CertHE)	0	1	0	1
	Level 3 (e.g. A level, AS level)	1	3	1	5
	Level 2 (e.g. GCSE, NVQ)	0	0	1	1
Occupation category (n)^a^	Executive	2	5	3	10
	Professional	7	8	14	29
	Technical support	6	4	2	12
	Sales	0	0	1	1
	Clerical	0	3	0	3
Yearly income (n)	£60,000+	2	3	3	8
	£40,000-59,999	2	3	4	9
	£20,000-39,999	12	12	11	35
	£0–19,999	0	2	2	4

*C* control group, *MA* mobile app group, *MA + SSWD* mobile app plus sit-stand work desk group, *HND* Higher National Diploma, *DipHE* Diploma of Higher Education, *CertHE* Certificate of Higher Education, *A Level* Advanced Level, *AS Level* Advanced Subsidiary Level, *GCSE* General Certificate of Secondary Education, *NVQ* National Vocational Qualification

^a^ There were no height or BMI measures for 2 control participants. One control participant did not provide an occupational category

### Recruitment and retention

**Fig. 3:**
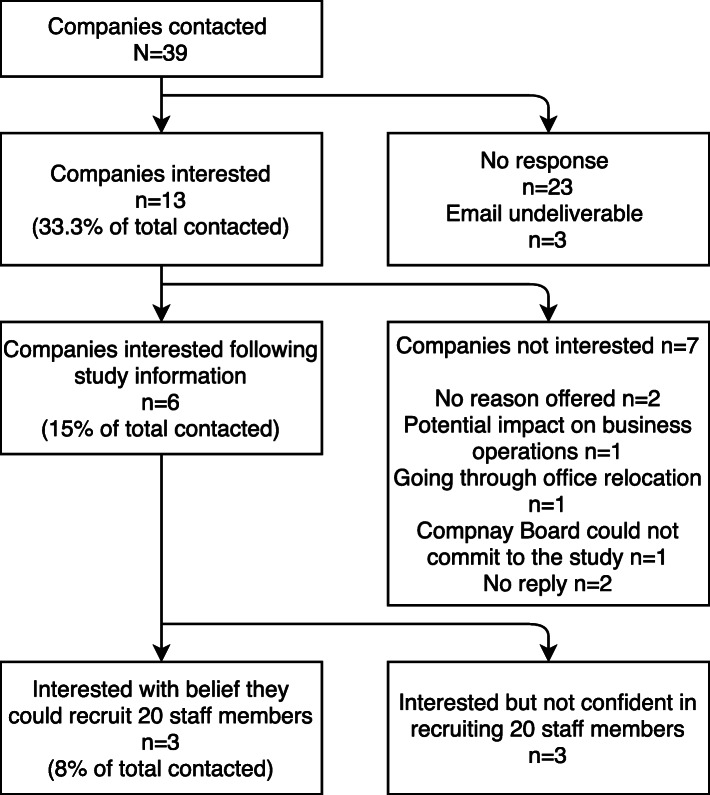
Recruitment of worksites

**Fig. 4:**
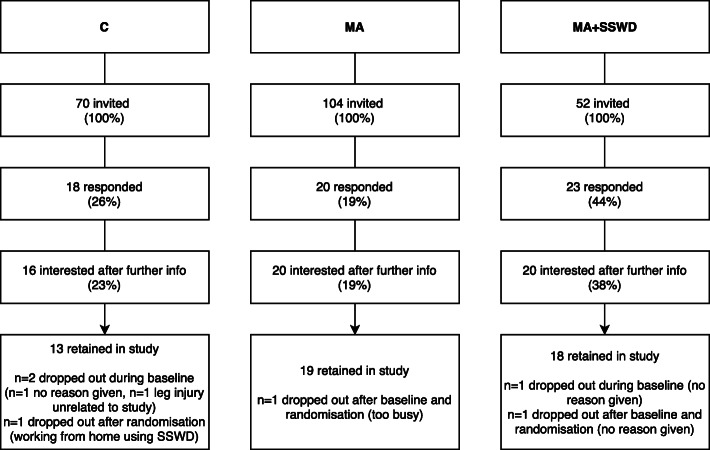
Recruitment and retention of individuals throughout the study

The recruitment of worksites and individual participants are documented in Figs. 3 and 4, respectively. A total of 39 companies across two cities in Northern Ireland were contacted via email, inviting them to participate in the intervention. After further information, six companies remained interested (15% of those approached). Of these, three did not believe they had the capability of recruiting 20 staff participants, therefore three companies were recruited (8% of those approached) (See Fig. 3). Within the three worksites, all employees were invited to participate. Both the MA and the MA + SSWD organisations were software companies and the control group were from a computer consultancy company, all based in Belfast, Northern Ireland. The MA company employed 104 staff, the MA + SSWD employed 52 staff and the control group employed 70 staff. Nineteen percent of the MA company, 44% of the MA + SSWD company and 26% of the control company responded with interest in participating (See Fig. 4). On provision of further information three people in the MA + SSWD and two in the control company decided not to participate. All of those who remained interested met inclusion criteria, consented and were recruited. This resulted in *n* = 20 (MA and MA + SSWD), and *n* = 16 (C). Recruitment was stopped at this point despite the target (*n* = 20 per group) not being met, due to project time constraints. In terms of retention, 95% of participants in the MA group, 90% in the MA + SSWD group, and 81% in the control group remained in the study until the end.

### Engagement

The Worktivity app sent hourly prompts reminding the participant to log their sitting time each working day. Over the course of the 8-week intervention, participants received a total of 336 prompts (42 days × 8 working hours). Participants in the MA group acknowledged the prompts to log sitting 66% of the time, more often than those in MA + SSWD (52%). They also responded to the prompts more quickly than the MA + SSWD; the mean response time for the MA group was 18 min, 22 s compared to 19 min, 32 s in the MA + SSWD group (Table 3). Insights from the exit survey suggest that several participants used the intervention as intended in the beginning, but as time went on they lost interest and disengaged.

**Table 3:** App prompt acknowledgement and response times

Group	Total prompts acknowledged n (%)	Timely responses (< 1 min) n (%)	Delayed responses (> 1 min) n (%)	Mean response times (min:ss)
MA	222 (66)	79 (35)	143 (65)	18:22 ± 7:07
MA + SSWD	173 (52)	63 (30)	111 (70)	19:32 ± 7:32
Overall	198 (59)	71 (33)	127 (67)	18:56 ± 7:14

Data are group means ± standard deviation where relevant. *MA* mobile app group, *MA + SSWD* mobile app plus sit-stand work desk group

### Delivery

All participants in the MA and MA + SSWD groups received a mobile phone with the Worktivity app installed and successfully set a ‘sit-less’ goal. All participants in the MA + SSWD group received the SSWD. Some responses from the exit survey suggest that parts of the intervention were not delivered as intended (Tables 4 and 5). Twenty-six percent of the MA group and 44% of the MA + SSWD agreed or strongly agreed that there were many technical issues with the app. For example, some participants reported that prompts to log sitting were issued at incorrect times and others reported the app crashing. There were also five instances where participants contacted the researcher to report issues with the timing of prompts, which led to the app being updated. Additionally, the educational elements were not always delivered as intended resulting in some participants (24%; *n* = 6 MA + SSWD, *n* = 3 MA) not recalling having ever received the educational facts/tips. The app was programmed to deliver the educational prompt at the end of each work day (after the eighth log) but in instances where data entry was not complete (i.e. logging sitting < 8 times), the educational prompt was not sent. Furthermore, if data entry was inaccurate the resulting feedback and prompts may also have been inaccurate. Some participants also noted issues with cabling which limited full extension of the SSWD. Two mobile devices had to be replaced due to charging issues. From the environmental audit, no environmental changes occurred that may have impacted intervention delivery (see Additional file 1).

**Table 4:** Responses to closed ended questions from the exit survey

Overall Programme MA (n = 20) MA + SSWD (***n*** = 18)	Strongly Agree (%)		Agree (%)		Neither agree nor disagree (%)		Disagree (%)		Strongly Disagree (%)	
	MA	MA+ SSWD	MA	MA+ SSWD	MA	MA+ SSWD	MA	MA+ SSWD	MA	MA+ SSWD
The programme was helpful in reducing my sitting time	5.00	16.67	30.00	55.56	35.00	22.22	30.00	0.00	0.00	5.56
I am likely to recommend the programme to a colleague	0.00	5.56	50.00	33.33	20.00	33.33	30.00	16.67	0.00	11.11
The programme is suitable for a workplace setting	20.00	16.66	45.00	55.56	15.00	11.11	20.00	16.67	0.00	0.00
I feel this intervention will have a lasting effect on reducing my sitting	5.26	5.56	47.37	22.22	10.53	44.44	31.58	22.22	5.26	5.26
I am satisfied with the overall intervention	5.26	5.56	52.63	33.33	26.32	33.33	15.79	16.67	0.00	11.11
The app helped me reduce my sitting	5.26	0.00	36.84	35.29	21.05	29.41	31.58	29.41	5.26	5.88
I am comfortable with using mobile app technology	42.10	72.22	57.90	22.22	0.00	0.00	0.00	0.00	0.00	5.56
The app is suitable for use in the workplace	0.00	0.00	73.68	33.33	15.79	16.67	10.53	38.89	0.00	11.11
It was easy to use the app	26.32	11.11	57.89	61.11	5.26	11.11	10.53	16.67	0.00	0.00
There were many technical issues with the app	5.26	11.11	21.05	33.33	26.32	11.11	42.11	22.22	5.26	22.22
I am likely to recommend the app to a colleague	0.00	0.00	57.89	5.56	10.53	38.89	21.10	27.78	10.53	27.78
Being able to set my own sitting goal was helpful	0.00	16.67	52.63	38.89	21.05	16.67	26.32	27.78	0.00	0.00
The reminders to self-report/log sitting time were too frequent	5.26	11.11	15.79	44.44	31.58	16.67	47.37	16.67	0.00	11.11
The reminders to self-report/log sitting were annoying	5.56	16.67	11.11	55.56	44.44	11.11	38.89	11.11	0.00	5.56
I responded to all of the reminders to self-report/log your sitting	0.00	0.00	31.58	22.22	10.53	11.11	36.84	33.33	21.05	33.33
The prompts to stand and move were helpful	0.00	0.00	42.11	38.89	31.58	27.78	21.05	16.67	5.26	16.67
The prompts to stand and move were annoying	0.00	11.11	10.53	38.89	36.84	38.89	42.11	11.11	10.53	0.00
After receiving a prompt to move/stand, I usually did	0.00	5.56	26.32	16.67	15.79	44.44	36.84	22.22	21.05	11.11
I am satisfied with how the app presented feedback and information	0.00	0.00	57.89	33.33	31.58	27.78	10.53	27.78	0.00	11.11
I would like to continue using the app after the study	0.00	0.00	0.00	0.00	31.58	5.56	42.11	33.33	26.32	61.11
I am satisfied with the app	0.00	0.00	57.89	16.67	26.32	27.78	5.26	27.78	10.53	27.78
The educational facts and tips were helpful	0.00	0.00	47.37	12.50	26.32	56.25	15.79	6.25	10.53	25.00
The educational facts and tips were repetitive	0.00	7.14	42.11	28.57	36.84	57.14	15.79	7.14	5.26	0.00
The educational facts and tips were annoying	0.00	7.14	0.00	28.57	36.84	57.14	52.63	7.14	10.53	0.00
I understood the information provided in the educational facts and tips	5.26	7.14	73.68	42.86	21.05	50.00	0.00	0.00	0.00	0.00
After reading the educational facts and tips, I actually applied them as well.	0.00	0.00	21.05	14.29	47.37	71.43	21.05	7.14	10.52	7.14
I am satisfied with the daily educational facts and tips	0.00	0.00	47.37	35.71	47.37	57.14	0.00	0.00	5.26	7.14
I am satisfied with the height adjustable desk	n/a	52.94	n/a	23.53	n/a	5.88	n/a	17.65	n/a	0.00
The height adjustable desk helped me reduce my sitting	n/a	58.82	n/a	17.65	n/a	5.88	n/a	11.76	n/a	5.88
I am comfortable with using a height adjustable desk at work	n/a	47.06	n/a	41.18	n/a	0.00	n/a	11.76	n/a	0.00
The height adjustable desk is suitable to be used in the workplace	n/a	47.06	n/a	35.29	n/a	0.00	n/a	17.65	n/a	0.00
It was easy to use the height adjustable desk	n/a	58.82	n/a	35.29	n/a	0.00	n/a	5.88	n/a	0.00
There were many practical issues using the height adjustable desk	n/a	5.88	n/a	11.76	n/a	11.76	n/a	41.18	n/a	29.41
I am likely to recommend the desk to a colleague	n/a	23.53	n/a	41.18	n/a	11.76	n/a	23.53	n/a	0.00
I would like to continue using the desk after the study	n/a	41.18	n/a	23.53	n/a	0.00	n/a	23.53	n/a	11.76
My productivity at work was affected negatively by participating in the programme	5.26	5.88	0.00	23.53	10.53	11.76	68.42	47.06	15.79	11.76
My productivity was affected negatively by receiving the reminders to log sitting throughout the day	0.00	0.00	10.53	41.18	15.79	23.53	57.90	29.41	15.79	5.88
My productivity was affected negatively by responding to the reminders to log sitting throughout the day	0.00	0.00	10.53	52.94	10.53	17.65	63.16	29.41	15.79	0.00
My productivity at work was affected negatively by using the height adjustable desk	n/a	0.00	n/a	11.76	n/a	0.00	n/a	52.94	n/a	35.29

*MA* mobile app group, *MA + SSWD* mobile app plus sit-stand work desk group

**Table 5:** Responses to open ended questions from the exit survey

Other	MA (%) (***n*** = 20)	MA + SSWD(%) (***n*** = 18)
Which aspect of the programme did you like best?	App self-monitoring 22.22	App self-monitoring 6.25
	App stand prompts 33.33	App stand prompts 37.50
	Educational tips 22.22	Educational tips 6.25
	Other 22.22	Other 50.00
Which aspect of the programme did you like least?	App self-monitoring 58.82	App self-monitoring 81.25
	App stand prompts 17.65	App stand prompts 6.25
	Educational tips 5.88	Educational tips 6.25
	Other 17.65	Other 6.25
What did you think about the intervention length?	Too short 5.56	Too short 5.56
	Too long 55.56	Too long 72.22
	Appropriate length 38.89	Appropriate length 22.22
What did you think about the amount of educational facts and tips you received?	Too few 16.67	Too few 10.00
	Too many 11.11	Too many 30.00
	Appropriate amount 72.22	Appropriate amount 60.00

*MA* mobile app group, *MA + SSWD* mobile app plus sit-stand work desk group

### Completion rates and usable data

At baseline, all participants provided demographic measures. In the control group, 13% did not provide their height and 6% did not provide an occupational category (Table 2). All workplaces agreed to an environmental audit being performed at baseline and follow-up. If not immediately obvious from the office layout, the company managers assisted with completing the audit data (e.g. providing information on flexible working policies and office temperature regulation). Table 6 summarises the valid and usable data obtained for the SB, productivity and mood outcomes. In terms of activPAL™ data for the overall day, across the three groups, valid and usable data were obtained from 88–95% of participants at baseline, 70–90% at mid-intervention and 69–80% at follow-up. The number of valid days of data in each group was high; no group mean at any time point was less than five days per seven-day data collection period. In terms of work day activPAL™ data (i.e. between 09:00–17:00), valid and usable data ranged from 81–95% at baseline, 69–90% at mid-intervention and 63–80% at follow-up. The number of valid days of data in each group was good; no group mean at any time point was less than three days per five-day data collection period.

**Table 6:** Completion rates and useable data

	Baseline			Mid-intervention			Follow-up		
	C	MA	MA+SSWD	C	MA	MA+SSWD	C	MA	MA+SSWD
Sedentary Behaviour (activPAL™)									
Participants with useable data (n)	14	19	18	13	18	14	11	16	16
Number of valid days	5.71 ± 1.49	6.11 ± 1.41	6.17 ± 1.29	5.08 ± 1.80	5.83 ± 1.38	6.43 ± 0.76	6.09 ± 1.45	5.94 ± 1.48	5.69 ± 1.62
% loss	12	5	10	19	10	30	31	20	20
Productivity (EMA)									
Participants with useable data (n)	14	20	19	13	19	17	13	19	17
Number of valid days	4.79 ± 0.43	4.85 ± 0.49	4.58 ± 0.69	4.92 ± 0.28	4.95 ± 0.23	4.42 ± 0.94	4.46 ± 0.97	4.37 ± 0.90	4.76 ± 0.44
% loss	12	0	5	19	5	15	19	5	15
Mood (BRUMS)									
Participants with useable data (n)	16	20	20	12	18	16	13	18	18
% loss	0	0	0	25	10	20	19	10	10

*Data are means±SD where relevant. C* control group, *MA* mobile app group, *MA + SSWD* mobile app plus sit-stand work desk group, *EMA* Ecological momentary assessment, *BRUMS* Brunel Mood Scale

In terms of productivity data, completion rates ranged from 88–100% at baseline and 81–95% at mid- intervention and follow up. The number of valid days of productivity data in each group was high; no group mean at any time point was less than four days per five-day data collection period. At baseline all participants provided BRUMS data. Completion rates ranged from 75–90% at mid-intervention and 81–90% at follow-up.

All measurement equipment was collected at the worksites by the researchers after each measurement period. No equipment was lost during the course of the study.

### Adverse events

Over the course of the study there were no serious adverse events, however there were a small number of minor issues with the activPAL™ devices. During the measurement periods there were 17 cases where participants recorded in their diaries that their activPAL™/adhesive patch caused some slight skin irritation. These 17 cases consisted of 13 individuals (*n* = 1 reported irritation at all three measurement points, *n* = 4 reported issues at two measurement points). There were six instances where participants removed the device due to the irritation. There were also ten identified cases of activPAL™ battery malfunction (i.e. battery did not hold charge for the full seven day measurement period).

### Acceptability

Tables 4 and 5 summarise the results from the satisfaction survey. The majority of participants from the MA group (65%) and the MA + SSWD group (72%) agreed that the intervention was suitable for integration into the workplace setting. However, overall satisfaction with the intervention was moderate to low; 58% of participants in the MA group strongly agreed or agreed that they were satisfied with the intervention compared to 39% in the MA + SSWD group. Similarly, 58% of those in the MA group strongly agreed or agreed they would recommend the intervention to a colleague, compared to only 6% of those in the MA + SSWD group. Nevertheless, 72% of those in the MA + SSWD group strongly agreed or agreed that the intervention was helpful in reducing their sitting, compared to 35% in the MA group.

Satisfaction levels with the height adjustable desk were high. Of those who received the SSWD, 76% strongly agreed or agreed that they were satisfied with the desk, 94% strongly agreed or agreed that the desk was easy to use and 65% strongly agreed or agreed they would like to use it after the intervention period. In terms of the mobile app, the majority of participants in both groups strongly agreed or agreed the mobile app was easy to use (MA 84%, MA + SSWD 72%). However, 26% of the MA group and 44% of the MA + SSWD felt that there were many technical issues with the app and no participants indicated they would be unlikely to use the app after the intervention ended. The most disliked feature in both groups was the app self-monitoring (MA 59%, MA + SSWD 81%).

## Discussion

This study explored the feasibility of delivering the Worktivity intervention, targeting SB reduction in desk-based office workers, in the workplace setting. Initial recruitment of companies to participate was challenging but retention of individual participants within the intervention was high. Office workers’ engagement with the intervention in terms of acknowledging and responding to app prompts (predominant intervention component), was moderate (on average 59%). All essential intervention elements were successfully delivered but due to technical issues not all behaviour change components were always delivered as planned e.g. educational tips. The amount and type of data collected during the study was appropriate, however instances of missing or unusable data (i.e. not meeting validity criteria) were observed with the activPAL™ data and data loss increased at each follow up time point. Although particiapnts indicated that the intervention was suitable for integration into the normal working day, overall satisfaction was moderate to low. Taken together, these findings suggest that, while in principle it is feasible to implement a SB mobile app intervention within the workplace setting, the technical feasibility of the Worktivity app was somewhat compromised and further refinements are needed before progressing to a larger effectiveness trial or implementation.

Recruiting companies to participate in this intervention was challenging but adherence and retention of individual participants was good. Initial recruitment of worksites was poor (8% of those approached) and within the recruited companies, recruiting individual participants was also somewhat challenging, resulting in the target sample size for this study not being met (93% of target recruited). However, participant retention within the study was high (81% C, 90% MA + SSWD, 95% MA). The same scenario has been reported in another feasibility study targeting workplace SB and using a similar email-based recruitment strategy [37]. Reflecting the results of this current study, it appears that establishing initial contact with organisations is one of the major barriers to recruitment into workplace health behaviour interventions. The research team had no contacts within any of the companies invited to participate. Additionally, with respect to SB, we have previously reported that company managers and/or employees do not necessarily see SB reduction as a priority or an issue that needs to be addressed [13]. Educating the target population on the importance of the behaviour change, establishing a good relationship with ‘gatekeepers’, and ‘selling’ the intervention well to the right people, all appear to be key to recruitment success. Another option to boost recruitment in the future is to offer a wait list control option to those who may be hesitant of allocation to the control group.

Engagement with the app prompts to self-monitor sitting time was moderate. Participants in the MA group acknowledged the prompts to log sitting more often than those in the MA + SSWD group (66% v 52%). This result is comparable to engagement levels found in another app based self-monitoring intervention to improve health behaviours (including SB reduction) in a sample of U.S. veterans [38]. It is possible that acknowledging and responding to prompts, or in other words, engagement with the self-monitoring element of the intervention, during working hours was compromised due to the prioritization of work-related tasks [13]. Furthermore, although prompts were delivered on an hourly basis to minimize self-reported SB recall bias, it is possible that the frequency of prompts eventually led to disengagement due to prompt ‘fatigue’ [39].

Although moderate engagement with the app prompts suggests that office workers can quickly self-report their sitting time using a mobile app, intervention designers may wish to consider even lower burden alternatives, for example using sensors and wearables, to measure SB in future studies.

In terms of intervention delivery, the app component of the intervention was not always implemented entirely as intended due to technical issues and non-compliance with self-reporting sitting time. Technical malfunctions appear to be common in digital health interventions [40, 41], and likely lead to disengagement [42]. It is therefore important that researchers do not underestimate the importance of rigorous laboratory testing and sufficient usability testing of digital interventions prior to implementation in a study, to fully address potential delivery issues and technical problems. It is also helpful to have IT support readily available throughout the intervention design, testing, and implementation phases in order to promptly resolve such issues. In addition to technical issues, some participants reported that they did not always log their sitting time hourly. Participants ignoring digital intervention content sent to encourage SB reduction has been reported elsewhere [37]. Mobile app usage tends to have a natural time course with evidence suggesting that mobile app usage is initially high but follows a sharp decline in as little as three weeks [43]. This highlights the engagement struggles that are inherent in mobile app interventions [44]. If extended engagement with a mobile app is intended, as it was with Worktivity through the self-monitoring component, it is essential to identify the optimal amount of intervention engagement for successful behaviour change whilst also addressing competing factors, in this case work productivity. Future work should also seek to identify indicators of engagement and the critical time point at which to re-intervene to prevent complete drop-out.

Sufficient amounts of valid, useable data on SB, productivity and mood were collected initially, however device-based SB data loss increased at each subsequent data collection time point. This suggests that quick, low-burden, EMA based assessments are a feasible method for use with office workers during the work day, but that alternative validated SB measurements methods may need to be considered. Particularly with respect to the activPAL™, our results suggest that data loss was due to device removal (skin irritation) and technical malfunctions. Skin irritation relating to skin mounted activPAL™ devices and adhesives has been reported elsewhere [36, 45]. Offering participants a variety of adhesive attachments to choose from and providing them with clear instructions such as online tutorials may be of benefit. The use of an elasticised pouch to secure the device instead of attaching it directly to the skin appears to be a valid and more practical method of attachment that could increase compliance [46]. Alternatively, participants could be advised to attach the activPAL™ to the opposite leg. Providing participants with a waterproof covering may increase activPAL™ weartime, so that they do not have to remove the device for bathing or swimming. Future qualitative work may help to uncover other methods to minimise data loss from activPAL™ non-compliance, but alternative device-based SB measurement tools that can accurately identify posture with minimal participant burden would be of benefit.

Finally, while participants agreed that the intervention could be implemented within the workplace setting, overall satisfaction with the Worktivity intervention was modest. Given the intervention was not fully delivered as planned due to technical issues and moderate levels of engagement with SB self-monitoring, lower than anticapted satisfaction is not surprising. It is also worth noting that the Worktivity app is a research-grade prototype that has not yet gone through the level of user experience testing and development that other highly funded commercial products receive. Therefore, some expectation mismatch regarding the interface and functionality of the app may have occurred. The ability to deliver evidence-based, satisfying and engaging behaviour change interventions via smartphones remains an ongoing challenge.

### Strengths and limitations

Outcome focused studies thus far dominate the SB research area [47] and preliminary evaluation has been largely overlooked in the development and testing of SB-reduction interventions to date [48]. The results of this feasibility study begin to address this gap by providing answers around the “how” and “why” Worktivity, and indeed other mHealth interventions, might be implemented successfully in a workplace setting. A further strength of this study is that the intervention was based upon behaviour change theory, was developed using an iterative participatory approach [22], and was specifically designed to be used in a real-world setting, thereby providing a robust basis from which to make refinements based on the findings from this study. There are, however, some limitations to the study. This sample was drawn from an educated white-collar workforce from one area in Northern Ireland and we excluded pregnant woman and non-ambulatory individuals from the study, therefore our findings may not be generalisable to a typical workforce or representative of the population that might be targeted for a future trial. Furthermore, given the nature of the trial, it was also not possible to blind the participants and assessors. However, the use of device-based measures (ActivPAL™) and participant reported outcomes of satisfaction, mood and productivity minimise researcher bias.

## Conclusions

The findings of this study suggest that, in principle, it is feasible to implement a mobile app-based intervention in the workplace setting however the Worktivity intervention requires further technical refinements prior to implementation. Engagement with the intervention may be improved by using more passive data collection via a smartphone or wearable sensors thereby reducing participant burden. Although the activPAL™ is a valid and reliable SB measurement tool, we observed levels of data loss which could impact future effectiveness trials, therefore alternative SB measurement approaches may be warranted. The most challenging aspect was the initial recruitment of worksites willing to participate and use this type of intervention. Educating employers and raising awareness at relevant forums on the benefits of sedentary behaviour reduction in the workplace is needed to stimulate interest and investment in future interventions. By overcoming these challenges, we believe that this type of digital intervention would be feasible to use with office workers in the workplace setting.

## Supplementary Information

**Additional file 1.** Environmental Audit.

## Data Availability

The datasets used and/or analysed during the current study are available from the corresponding author on reasonable request.
