# Supplementary material for: The “Worktivity” mHealth intervention to reduce sedentary behaviour in the workplace: a feasibility cluster randomised controlled pilot study
Source: BMC Public Health. 2021 Jul 18;21:1416. doi: 10.1186/s12889-021-11473-6 (PMC8286585; doi:10.1186/s12889-021-11473-6)
Supplement: Supplementary file 1 — Additional file 1. Environmental Audit. [file 12889_2021_11473_MOESM1_ESM.docx]

Additional File 1. Environmental Audit

|  | **C** | **MA** | **MA+SSWD** |
| --- | --- | --- | --- |
| Is the office open plan? |  |  |  |
| Are the desks communal? |  |  |  |
| Are the desks booth-like? |  |  |  |
| Do staff have their own offices? |  |  |  |
| Are the offices centralised to one area? |  |  |  |
| Is there the opportunity to stand in the office? |  |  |  |
| Do staff have their own desktop PC? |  |  |  |
| Do staff have access to a work laptop? |  |  |  |
| Do staff have access to their own laptop? |  |  |  |
| Do staff have access to mobile devices such as I pads/tablets? |  |  |  |
| Do staff have a work mobile phone? |  |  |  |
| Are staff permitted to use their own mobile at work? |  |  |  |
| Do staff have a work landline on their desks? |  |  |  |
| Do staff have access to a cordless landline? |  |  |  |
| Do staff have access to a headset for phone calls? |  |  |  |
| Do staff have their own printer at their desk? |  |  |  |
| Does the office have communal printers? |  |  |  |
| Does the office have communal photocopiers? |  |  |  |
| Do staff have their own bin at their desk? |  |  |  |
| Does the office have communal bins? |  |  |  |
| Do staff have their own filing/storage space at their desk? |  |  |  |
| Does the office have communal filing/storage space? |  |  |  |
| Is there/ are there elevators in the building? |  |  |  |
| Can the offices be accessed by elevator? |  |  |  |
| Are there stairs in the building? |  |  |  |
| Can the offices be accessed by stairs? |  |  |  |
| Is there a central canteen/kitchen in the building? |  |  |  |
| Is there a kitchen or break room for staff? |  |  |  |
| Are the tables and chairs in the kitchen/break room? |  |  |  |
| Is there the opportunity to stand in kitchen/break room? |  |  |  |
| Are there tables and chairs in the meeting room? |  |  |  |
| Is there the opportunity to stand in the meeting rooms? |  |  |  |
| Are there shower/changing facilities in the workplace |  |  |  |
| Are the lights controlled by movement sensors? |  |  |  |
| Is temperature regulated to remain constant? |  |  |  |
| Do staff have flexible working/lunch hours? |  |  |  |

C= control group, MA= mobile app group, MA+SSWD= mobile app plus sit-stand work desk group; Green=Yes, Red=No, Yellow=Some
